# The prevalence of *Caenorhabditis elegans* across 1.5 years in selected North German locations: the importance of substrate type, abiotic parameters, and *Caenorhabditis* competitors

**DOI:** 10.1186/1472-6785-14-4

**Published:** 2014-02-06

**Authors:** Carola Petersen, Philipp Dirksen, Swantje Prahl, Eike Andreas Strathmann, Hinrich Schulenburg

**Affiliations:** 1 Department of Evolutionary Ecology and Genetics, Zoological Institute, Christian-Albrechts University, Am Botanischen Garten 1-9, 24118 Kiel, Germany

**Keywords:** *Caenorhabditis elegans*, *Caenorhabditis remanei*, Niche separation, Competition

## Abstract

**Background:**

Although the nematode *Caenorhabditis elegans* is a major model organism in diverse biological areas and well studied under laboratory conditions, little is known about its ecology. Therefore, characterization of the species’ natural habitats should provide a new perspective on its otherwise well-studied biology. The currently best characterized populations are in France, demonstrating that *C. elegans* prefers nutrient- and microorganism-rich substrates such as rotting fruits and decomposing plant matter. In order to extend these findings, we sampled *C. elegans* continuously across 1.5 years from rotting apples and compost heaps in three North German locations.

**Results:**

*C. elegans* was found throughout summer and autumn in both years. It shares its habitat with the related nematode species *C. remanei*, which could thus represent an important competitor for a similar ecological niche. The two species were isolated from the same site, but rarely the same substrate sample. In fact, *C. elegans* was mainly found on compost and *C. remanei* on rotten apples, possibly suggesting niche separation. The occurrence of *C. elegans* itself was related to environmental humidity and rain, although the correlation was significant for only one sampling site each. Additional associations between nematode prevalence and abiotic parameters could not be established.

**Conclusions:**

Taken together, our findings vary from the previous results for French *C. elegans* populations in that the considered German populations always coexisted with the congeneric species *C. remanei* (rather than *C. briggsae* as in France) and that *C. elegans* prevalence can associate with humidity and rain (rather than temperature, as suggested for French populations). Consideration of additional locations and time points is thus essential for full appreciation of the nematode's natural ecology.

## Background

The nematode *Caenorhabditis elegans* has become one of the main model organisms in biological research, where it proved of extreme value in diverse disciplines such as animal development and genetics, neurobiology and behaviour, and genome architecture and function [1]. Several specific characteristics have contributed to the nematode’s popularity over the last decades, for example its known genome, its short generation time, the ease of obtaining isogenic lines by selfing, and the possibility to produce frozen stocks. Intriguingly, however, almost all research with this worm is done with a single wildtype strain, called N2, which has been kept in the laboratory for several decades and is known to have specifically adapted to the commonly used experimental conditions [2,3]. In contrast, only very little is known about the nematode’s natural environment and ecology, including its life-history in the field, its seasonal abundance, global distribution, or association with other organisms. Such information may be of high value for our understanding of *C. elegans* biology: the exact and true function of numerous genes, many of which are still of unknown function, may only be revealed if the species' natural conditions are taken into account.

Over the last couple of years, an increasing number of studies began to assess different aspects of the ecology of this nematode, yielding a first overview of *C. elegans* in its natural habitat. *C. elegans* has been found in most parts of the world [4], although only rarely in the tropics [5]*.* In temperate regions, it is common and often coexists with its congeneric relatives *C. remanei* and *C. briggsae*[5]. In the past, *C. elegans* was usually considered to inhabit soils [6-8], although even then it was already known from other substrates such as decaying mushrooms, leaf litter, garden compost [4,9], or invertebrates [10-14], usually in anthropogenic environments [15]. More recent studies focused on compost heaps and rotten fruits and showed that proliferating *C. elegans* populations can be found regularly in these substrates [6,12,14,16,17]. The predominant stage of *C. elegans* found in compost heaps is the dauer stage. Recently rotten fruits and decomposing plant stems were found to contain populations of all stages including populations without any dauer larvae [17].

The aim of our study was to investigate the population dynamics of *C. elegans* and other *Caenorhabditis* species at different locations in Northern Germany across time. We additionally aimed at evaluating in how far the presence of *C. elegans* is related to abiotic parameters such as atmospheric temperature, which is expected to affect fitness of ectotherms [18,19], including *C. elegans* under laboratory conditions [20,21]. Furthermore, we also considered additional environmental parameters that may affect nematode proliferation such as the amount of rain fall, environmental humidity and pH. In order to identify suitable substrates for such long-term analyses, we initially screened high numbers of decaying mushrooms, different parts of *Arum maculatum*, and a variety of decomposing fruits, plants as well as compost samples from different locations in Northern Germany. Compost and rotten apples contained *Caenorhabditis* most frequently. Therefore, for the current study, we specifically focused our sampling efforts on these two substrates. Here, we now present our results from 18 months continuous sampling of compost and rotting apples from three North German locations.

## Methods

### Sampling sites and types of samples

Sampling was carried out continuously once to twice every month from July 2011 to December 2012. We focused on three North German locations (Kiel, Münster, and Roxel) and two substrate types (compost, rotten apples), which we found in initial pilot studies to contain high frequencies of *Caenorhabditis* nematodes. In Kiel the samples were collected in the botanical garden (54°20'N and 10°06'E) from three large compost heaps and additionally from rotten apples collected from a locally separated apple heap and from below nearby apple trees. In Münster, samples were collected in a meadow of the city's farming museum (51°56'N and 7°36'E) where a compost heap and apple trees are found in close vicinity. Roxel (51°57'N and 7°32'E) is a village close to Münster, where the samples were taken from three small compost heaps in a private garden.

Two substrate types were collected (compost and rotting apples). Compost was sampled from all three locations. In Kiel three big compost heaps ranging from fresh to old compost were used. The fresh compost consisted of plant material including grass, leaves, parts of bushes, parts of various plants and soil. The old compost consisted mainly of soil, but also contained some plant material. The compost heaps were mixed three times during the sampling period, once each in October 2011, May 2012, and October 2012. In Münster there was one compost heap on a meadow. The composition was similar to the fresh compost in Kiel, although it additionally contained straw and feces of sheep. This compost was not stirred during the considered sampling period. The compost in Roxel consisted of three small heaps of different ages in mesh cages. The fresh compost with mainly plant parts but also some organic garbage from the owners was only slightly degraded, whereas the older composts consisted mainly of soil and remaining parts of leaves and trees. The Roxel composts were stirred and mixed exactly once during the sampling period, namely in May 2012. In Kiel and Münster, rotting apples were additionally available. In Kiel, rotten apples could be sampled during the entire year from a specific apple compost heap. In Münster, the number of apples was limited, as they were left to rot under the trees in a meadow, on which sheep were kept continuously.

### Collection of samples and isolation of nematodes

Compost samples were taken from up to 15 cm depth of the compost heaps. Rotten apples were picked up from the ground. Compost and apples were collected in plastic bags. For most months and especially for the compost heaps, we collected at least six independent samples per site, and the distance between each of these was at least 50 cm for the Roxel compost and at least 100 cm for all other sites, ensuring at least some independence of the samples from a particular location and day. All samples were kept at approximately 10°C until they were processed in the laboratory. Sample processing was completed within 24 hours after collection to minimize changes in the present nematode developmental stages and also in the species composition of associated organisms. Approximately 5 g compost material or alternatively four to five apple pieces were distributed around a spot of 150 μl of an overnight culture of *Escherichia coli* OP50 on 9 cm peptone free medium (PFM) agar plates [22]. The nutrient-limited PFM agar was used to ensure standardized conditions across sampling dates and substrate types, because it minimizes growth of naturally associated microbes. As these naturally associated microbes are likely to vary across time and substrate and as they may also differ in their ability to grow on the standard nutrient-rich laboratory growth media, usage of such nutrient-rich media could lead to a substantial change in microbial composition and in turn nematode abundance. For each of the collected samples (e.g., a particular apple or a particular compost soil sample) we prepared exactly one sampling plate. The plates were checked for the presence of worms using a dissecting scope. Nematodes resembling *Caenorhabditis* were transferred individually onto new 6 cm PFM plates within the first five hours using a platinum picker. The plates were again checked for nematodes after 24 hours and after three to seven days. With few exceptions, we transferred no more than ten individual worms per sample. Based on this sampling scheme, we considered the worms isolated from different samples to be independent findings for a particular location, day, and substrate type, while the worms from one specific sample may be of common origin. We still assessed several worms per sample, in order to test whether different *Caenorhabditis* species coexist in one piece of substrate and are thus likely to interact with each other in nature.

### Species identification using microscopy and PCR

The isolated worms were kept for several days on plates. Further consideration of nematodes was based on the following criteria (see also previous *Caenorhabditis* sampling approaches in [6,12,17,22]): (i) Nematodes should produce offspring; (ii) they should show morphological features characteristic for *Caenorhabditis*[22]; and (iii) they should test positive in diagnostic genus- or species-specific PCRs [6,22]. For the latter, DNA of *Caenorhabditis* candidates was isolated by transferring one to five worms into 20 μl 1× PCR-buffer, including 1 μl Proteinase K (10 mg/ml). Worms were frozen for at least one hour at −80°C to break up tissue, digested for one hour at 50°C and boiled for 15 minutes at 95°C in a PCR machine (biolab products, LabCycler). For the species-specific primer pairs (Table 1), 1 μl of the DNA was subjected to the following reaction conditions: 95°C for 2 min followed by 35 cycles of 95°C for 1 min, primer-specific annealing temperatures for 0.5 min (Table 1) and 72°C for 1 min, and at the very end a final extension step at 72°C for 10 min. Conditions for *Caenorhabditis* genus-specific primers were 95°C for 2 min followed by 40 cycles of 95°C for 1 min, 70°C for 0.75 min and 72°C for 2.25 min, and additionally a final extension step at 72°C for 10 min (Table 1). The exact amplification conditions for all species-specific PCRs and also the primers specific for *C. elegans* and *C. remanei* were optimized by us to ensure unequivocal identification of the three most commonly encountered *Caenorhabditis* taxa (Additional file 1: Figure S1). For *C. elegans*, species identity was confirmed using two diagnostic primer pairs: One targeting a variable part of the recently duplicated immunity gene *nlp-30*, which is exclusively present in *C. elegans*[23], and the other targeting the *zeel-1*/*peel-1* compatibility locus [24]. Species identity was further verified for 21 randomly chosen *C. elegans* and 16 randomly chosen *C. remanei* natural isolates using crossing experiments with males from characterized laboratory strains. For 24 randomly chosen *C. briggsae* isolates, species identity was additionally confirmed by Sanger sequencing of the ribosomal ITS2 region, using the primers of the *Caenorhabditis*-diagnostic PCR (Table 1). The initial plates that contained worms tested positive for *Caenorhabditis* were subsequently used to bleach and freeze these strains for future studies.

**Table 1:** **Diagnostic primers used for identification of *Caenorhabditis *** **species or genus**

**Primer name**	**Primer sequence 5’-3’**	**Anneal T**^**a**^	**Target region**	**Specificity**	**Size**^**b**^	**Reference**
nlp30-F	ACACATACAACTGATCACTCA	55°C	*nlp-30*	*C. elegans*	154 bp	This study
nlp30-R	TACTTTCCCCATCCGTATC					
zeel/peel-leftF	CTGAAGCATGCCGGATTTAT	59°C	Region with *zeel-1* + *peel-1*	*C. elegans*	940 bp	[24] and this study
zeel/peel-leftR	TCCGTCCAATATTCAATCGAC					
Cre-ITS2-F1	TTGTCGGGCGGCATTGGGGCT	65°C	Ribosomal ITS2	*C. remanei*	300 bp	This study
Cre-ITS2-R4	CGTCGTCTTCCTTACCCCGAA					
Cbriggsae-F	GAACCTGCGAGTGCATG	56°C	*glp-1*	*C. briggsae*	302 bp	[22]
Cbriggsae-R	CCGTCTGCAAACGAACGGGC					
KK5.8S-1	CTGCGTTACTTACCACGAATTGCARAC	70°C	Ribsomal ITS2	*Caenorhabditis*	2008 bp	[14]
KK28S-4	GCGGTATTTGCTACTACCAYYAMGATCTGC					

^a^Optimized annealing temperature for diagnostic PCR.

^b^PCR product size.

For an analysis of proliferating nematode populations, we only considered worms, which were isolated and transferred individually to separate plates within five hours after sample processing (see above). At this point, developmental stage of the worms was scored. Thereafter, the worms were allowed to reach adulthood. If the adult worms showed the morphological characteristics of *Caenorhabditis*, they were subjected to the diagnostic PCR tests. The final results table only included worms belonging to the *Caenorhabditis* genus.

### Measurement of abiotic variables

Atmospheric temperature and humidity were recorded directly during sampling with a digital multi-purpose thermo-hygrometer (TFA Dostmann GmbH & Co. KG). The measurement was done directly above the collected substrates in a distance of 5 to 10 cm. The pH of collected compost and apples was measured in the laboratory using a mobile pH-meter (PCE-228, PCE Deutschland GmbH). For this purpose compost samples were mixed in the plastic bag and the electrode was brought into direct contact with the sample. In the rotten apples the measurement was done in an apple part that represented a decomposition stage similar to the pieces used for worm isolation. Additionally the average amount of rain per month for Kiel and Münster was obtained from Germany’s National Meteorological Service, the Deutscher Wetterdienst (DWD). As Münster and Roxel are close to each other, the data for rain in Münster was taken for both locations.

### Statistics

The current study explores *Caenorhabditis* occurrence across time in different substrates and locations. Due to the time-consuming sampling scheme, we did not include replication for a particular substrate type and location. Temporal dynamics in species prevalence is thus summarized using absolute numbers and percentages. Two types of statistical tests were applied with caution for each a particular substrate type and location separately, in order to (i) assess overall variation in species prevalence, and (ii) relate the proportion of nematode-containing samples with any of the measured abiotic parameters. For the first type of analyses, we used frequency tests, namely the Fisher exact test, for pairwise comparisons of species abundance (number of independent samples containing a particular species) across the entire sampling period and for each substrate type and location separately. For the second type of analyses, we assessed the presence of correlations between the proportion of samples with either *C. elegans* or *C. remanei* and each of the measured abiotic factors temperature, humidity, rain and pH, using a Spearmans-rank-test (for each substrate type and location separately). Only data sets with at least five sampling months in a row were taken into account for the correlation analysis. All statistical tests were performed with the program JMP version 9.0.2 (SAS Institute Inc.). For each of the analyses, multiple testing was accounted for by adjusting the significance level using the false discovery rate (FDR; [25]). Graphs were produced with R version 2.15.2 and Microsoft Office Excel 2007.

## Results

### *Caenorhabditis* species diversity and substrate association in Northern Germany

We assessed a total of 663 independent samples for presence of *Caenorhabditis* nematodes (498 × compost, 165 × rotten apple; Table 2; Additional file 2: Figure S2, Additional file 3: Figure S3, Additional file 4: Figure S4; Additional file 5: Table S1 and Additional file 6: Table S2). Based on our sampling scheme, we obtained isolates of three *Caenorhabditis* species, namely *C. elegans, C. remanei*, and *C. briggsae*, but no other *Caenorhabditis* taxon. The three species were found in different quantities, whereby *C. elegans* was generally most common. In detail, 149 of the collected 498 compost samples (30% of the 498 compost samples) contained *Caenorhabditis* nematodes. At all sampling sites, compost samples contained significantly more often *C. elegans* than *C. remanei* or *C. briggsae* (Table 3). 63 out of the 165 collected rotten apple samples (38% of the 165 apples) harboured *Caenorhabditis* worms*.* For the apples from Kiel, *C. remanei* was significantly more common than *C. elegans* or *C. briggsae* (Table 3)*.* Surprisingly, *C. elegans* was never found on rotten apples in Kiel. Although both compost and rotten apples in general contained each of the three species, coexistence of at least two species was only occasionally observed: Five compost samples (1% of the total 498 compost samples) contained both *C. elegans* and *C. remanei*, while a different set of five compost samples (1%) harboured both *C. elegans* and *C. briggsae. C. remanei* and *C. briggsae* were never isolated from the same substrate sample.

**Table 2:** Total number of independent samples and overall species prevalence

**Origin**	**Total samples**^**a**^	***C. elegans***		***C. remanei***		***C. briggsae***	
		**Positive samples**^**b**^	**Ind**^**c**^	**Positive samples**	**Ind.**	**Positive samples**	**Ind.**
Kiel apple	123	0 (0)	0	41 (0.33)	173	0 (0)	0
Münster apple	42	6 (0.14)	32	10 (0.24)	42	6 (0.14)	15
Kiel compost	170	30 (0.18)	114	7 (0.04)	23	5 (0.03)	12
Münster compost	129	27 (0.21)	124	1 (0.01)	6	4 (0.03)	10
Roxel compost	199	70 (0.35)	348	4 (0.02)	5	1 (0.01)	1

^a^Total number of independent samples per substrate type and location.

^b^Number and proportion (in brackets) of independent samples that contained the respective *Caenorhabditis* species.

^c^Total number of individuals isolated for the respective species from the indicated substrate type and location.

**Table 3:** **Fisher Exact test on the variation of species abundance per substrate type and location**^**a**^

**Origin**	**CE vs. CR**	**CE vs. CB**	**CR vs. CB**
**Kiel apple**	<0.0001*	>0.99	<0.0001*
**Münster apple**	0.4052	>0.99	0.4052
**Kiel compost**	<0.0001*	<0.0001*	0.7703
**Münster compost**	<0.0001*	<0.0001*	0.3701
**Roxel compost**	<0.0001*	<0.0001*	0.3718

^a^The frequencies used for this analysis are given in Table 2. The three Caenorhabditis species are compared pairwise. *Abbreviations*: *CEC. elegans*, *CRC. remanei*, *CBC. briggsae*. The false discovery rate was used to account for multiple testing and the remaining significant *P*-values (at α < 0.05) are given in bold and indicated by an asterisk.

### Seasonal abundance

**Figure 1:**
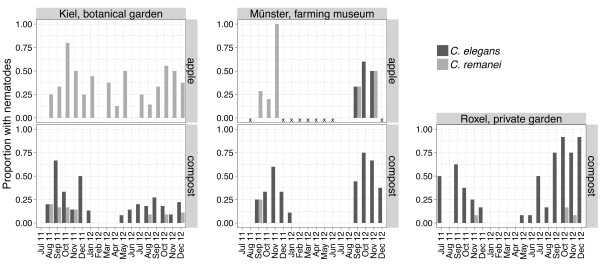
**Occurrence of *C. elegans *and *C. remanei *on compost and apple samples between July 2011 and December 2012 at different sampling sites.** The months for which no rotten apples were available in the Münster farming museum are marked with a cross. In Kiel, rotten apple samples could be collected during the entire sampling period. For each substrate and location, at least six and usually more than ten independent samples were assessed for the presence of *Caenorhabditis* nematodes. More details are in given in Table 2, and Additional file 5: Table S1 and Additional file 6: Table S2.

Compost could be collected all year long at all sampling sites and contained *Caenorhabditis* worms between July 2011 and January 2012 and between May 2012 and December 2012 (Figure 1; Additional file 6: Table S2). *C. elegans* was most abundant in Kiel compost in September 2011, in Münster compost in October 2012, and in Roxel compost in September and December 2012. Overall late summer and autumn seem to be the time periods with highest *C. elegans* prevalence (Figure 1). *C. remanei* was found in compost from August to November 2011, in August 2012 and from October to December 2012. This species showed highest abundance in Kiel in August 2011, in Münster in September 2011 and in Roxel in October 2012. *C. briggsae* was only rarely found, with peak frequencies in Kiel compost in August 2011 and in Münster compost in October 2011. In Roxel *C. briggsae* was only found once in August 2012.

Rotten apples were available in Kiel all year long and in Münster always from July to November. *C. elegans* was only found on rotten apples in Münster from September to November 2012 with highest abundance in October 2012 (Figure 1; Additional file 6: Table S2). *C. remanei* was found on rotten apples continuously between August 2011 and December 2012. Peak frequencies were recorded for Kiel in October 2011 and for Münster in November 2011 (Figure 1). *C. briggsae* was only found on apples in Münster, where it was common in October 2011.

### Variation in the presence of proliferating *Caenorhabditis* populations

After individual collection of samples, these were kept at approximately 10°C up to 24 hours before analysis on PFM plates. Within one to five hours after transfer onto plates, the samples were specifically assessed for indications of proliferating nematode populations, for example the presence of stages other than the long-lasting dauer stage. In the case of *C. elegans*, proliferating populations were found in several compost samples from all three locations, especially Roxel compost and Münster apples (Table 4). For *C. remanei*, proliferating populations were identified for rotten apples, especially from Kiel, and also compost (Table 4). *C. briggsae* was always isolated as a dauer stage during the early one to five hours-period.

**Table 4:** **Proliferating *Caenorhabditis *** **populations: Number of independent *Caenorhabditis*-containing samples and worms isolated within few hours upon substrate processing**

**Origin**	***C. elegans***				***C. remanei***				***C. briggsae***			
	**Samples**^**a**^		**Individuals**^**b**^		**Samples**		**Individuals**		**Samples**		**Individuals**	
	**Total**^**c**^	**Non-D**^**d**^	**Total**^**c**^	**Non-D**^**d**^	**Total**	**Non-D**	**Total**	**Non-D**	**Total**	**Non-D**	**Total**	**Non-D**
Kiel apple	0	0	0	0	15	15	54	40 (7, 2, 31)	0	0	0	0
Münster apple	4	4	12	7 (3, 1, 3)	3	3	8	8 (0, 0, 8)	3	0	3	0
Kiel compost	21	5	75	6 (6, 0, 0)	2	2	11	11 (3, 1, 7)	3	0	9	0
Münster compost	18	5	94	10 (3, 1, 6)	0	0	0	0	2	0	3	0
Roxel compost	48	20	169	36 (14, 4, 18)	3	3	4	4 (1, 0, 3)	0	0	0	0

^a^Number of independent samples from which *C. elegans, C. remanei* or *C. briggsae* were isolated within few hours after sample processing.

^b^Number of individual worms isolated within few hours after sample processing.

^c^Total numbers irrespective of developmental stage.

^d^Numbers for only non-dauer stages with further details given in brackets in the following order: L1-L3, L4, adult.

### The influence of abiotic factors on the occurrence of *C. elegans* and *C. remanei*

**Figure 2:**
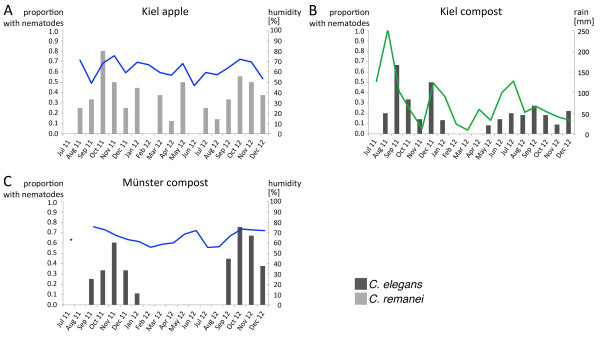
**Significant correlations between abiotic factors and proportion of samples containing *C. elegans *(dark grey bars) or *C. remanei *(light grey bars).** Humidity (blue lines) correlates with the proportion of rotten apple *C. remanei*-containing samples in Kiel **(A)** and with the *C. elegans*-containing proportion of compost samples in Münster **(C)**. Rain (green line) correlates with *C. elegans* prevalence in Kiel compost samples **(B)**. The results of the correlation analysis are given in Tables 5 and 6.

The correlation between the occurrence of *C. elegans* and *C. remanei* and the abiotic factors atmospheric temperature, humidity, rain and pH was analyzed for each sampling site and substrate type separately. The Spearman’s rank test showed a significant positive correlation of humidity with, on the one hand, the proportion *C. elegans*-containing compost samples from Münster and, on the other hand, the proportion of *C. remanei*-containing apple samples from Kiel (Figure 2, Tables 5 and 6). Additionally, in Kiel rain was correlated positively with *C. elegans* prevalence in compost (Figure 2, Table 5). *C. elegans* prevalence also correlated negatively with temperature in Münster apples, although in this case correlations were based on only few sampling dates and thus possibly unreliable (Table 5). None of the other considered correlations was significant or showed at least a statistical trend (Tables 5 and 6).

**Table 5:** **Correlations between mean proportion of *C. elegans*-containing samples and abiotic factors**

**Location**	**Substrate**	**Correlating abiotic factor**	***Rho***^***a***^	**N**	***P***^***b***^
Kiel	Compost	Temperature	0.2203	17	0.3955
		pH	−0.1072		0.8328
		Humidity	0.0069		0.8881
		Rain	0.1580		0.0060*
Münster	Apple	Temperature	−0.8416	9	0.0044*
		pH	0.6337		0.0669
		Humidity	0.5446		0.1295
		Rain	0.0594		0.8793
Münster	Compost	Temperature	−0.4596	17	0.0634
		pH	−0.1683		0.5184
		Humidity	0.6296		0.0068*
		Rain	0.1463		0.5753
Roxel	Compost	Temperature	0.1299	17	0.6193
		pH	−0.2845		0.2684
		Humidity	−0.2684		0.2976
		Rain	0.2882		0.2620

^a^Correlations were assessed with a non-parametric Spearman's rank test.

^b^Significance levels were corrected for multiple comparisons using the false discovery rate; the resulting significant correlations at α < 0.05 are given in bold and indicated by an asterisk.

**Table 6:** **Correlations between mean proportion of *C. remanei*-containing samples and abiotic factors**

**Location**	**Substrate**	**Correlating abiotic factor**	***Rho***^***a***^	**N**	***P***^***b***^
Kiel	Apple	Temperature	−0.1394	17	0.5936
		pH	−0.1727		0.5073
		Humidity	0.6179		0.0082*
		Rain	−0.3702		0.1436
Kiel	Compost	Temperature	0.1869	17	0.4726
		pH	−0.1072		0.6822
		Humidity	0.0069		0.9791
		Rain	0.1580		0.5447
Münster	Apple	Temperature	−0.4265	9	0.2523
		pH	0.0783		0.8412
		Humidity	0.3917		0.2972
		Rain	−0.5919		0.0932
Münster	Compost	Temperature	0.1531	17	0.5575
		pH	−0.4082		0.1038
		Humidity	0.4085		0.1035
		Rain	0.0510		0.8458
Roxel	Compost	Temperature	−0.1937	17	0.4563
		pH	−0.1365		0.6013
		Humidity	−0.1882		0.4695
		Rain	−0.2491		0.3350

^a^Correlations were assessed with a non-parametric Spearman's rank test.

^b^Significance levels were corrected for multiple comparisons using the false discovery rate; the resulting significant correlations at α < 0.05 are given in bold and indicated by an asterisk.

## Discussion

We here provide one of the few long-term ecological studies on *C. elegans*, one of the main model species in biological research. Our study covers an 18-month period and three independent sampling sites, thus yielding a broader perspective on *C. elegans* ecology than most previous studies [6,12,14,16,17]. Similar ecological information is rare for most of the classical genetic model taxa like yeast, *Drosophila melanogaster* or mice - in spite of its potential importance for in-depth understanding of the comprehensive laboratory-derived information on the species' biologies.

### High prevalence of *C. elegans* and *C. remanei* in Northern Germany

Our results generally confirm the recent study from mainland France that the diversity of *Caenorhabditis* species seems to be low at individual locations, at least in Central Europe [17], in contrast to the higher taxon diversity observed for a tropical rainforest in French Guiana [26]. Across the three North German sampling sites, we mainly found *C. elegans* and *C. remanei*, whereas *C. briggsae* was only occasionally found in small numbers. Other *Caenorhabditis* species could not be detected. Curiously, *C. remanei* appears to be restricted to Germany and neighboring regions (e.g., Elsace in Eastern France) [15,17], even though at a global scale it is found across a variety of temperate locations, for example in North America and Japan [15]. One possible reason for the species limited distribution in Europe is competition with a species with a similar ecological niche. Consistent with this idea, the congeneric *C. briggsae* seems to be very common in Europe - except for the German locations with *C. remanei*. This is best documented for the comprehensively sampled locations in France, revealing high prevalence of *C. elegans* and *C. briggsae*, but absence of *C. remanei*[17]. The *Caenorhabditis* species may additionally compete with other nematode taxa, which were regularly seen (yet not further characterized) in the same substrate samples.

### Substrate preference of different *Caenorhabditis* species

In general *C. elegans* may show a hemerophile life-style and thus occurs more frequently in places influenced by humans [15]. Such anthropogenic habitats probably provide a more constant supply of nutrient-rich organic material and thus microbial food throughout the year. Curiously, in previous studies, *C. elegans* was most often found as dauer with no reproductive activity in compost heaps [12,27]. Even though dauers were usually the most frequent stage in our study, a considerable proportion of samples did contain proliferating *C. elegans* populations (Table 3). It would be important in the future to evaluate which specific characteristics of the compost samples favour nematode proliferation.

*C. elegans* could not be isolated from any rotten apple from Kiel and was also absent from the 2011 apple collection from Münster. In 2012, however, this nematode was found on some of the Münster apples, in all cases with proliferating populations (Table 3). These particular apples were collected very close to the compost heap that harboured *C. elegans*. Therefore, it is possible that migration from the compost heap accounted for the nematode's presence on apples. In general, the rare occurrence of *C. elegans* on apples in Northern Germany contrasts with observations in France, where *C. elegans* is especially abundant on decaying apples [17]. At the same time, North German apples contained high frequencies of *C. remanei*, which was more or less absent from France [17]. Moreover, both species were occasionally isolated from the same substrate sample and are thus likely to interact directly with each other in nature. Therefore, it is conceivable that *C. remanei* may outcompete *C. elegans* on rotten apples, forcing *C. elegans* to use other substrate types in the North German locations.

### Influence of abiotic conditions on the occurrence of *Caenorhabditis* species

To obtain a first impression of the influence of abiotic parameters, we related the occurrence of *C. elegans* and *C. remanei* to temperature, humidity, rain fall, and pH. Temperature is likely of importance in this context [18,19], as previously isolated, natural *C. elegans* strains differ in their temperature preference, which appears to underlie natural variation in fitness [20,21]. Our current results do not support these previous observations, as we did not identify a positive association between atmospheric temperature and nematode occurrence. The potential influence of temperature may thus either be weak, obscured by additional factors, and/or missed by our approach, which did not allow us to obtain measurements at the micro-scale, possibly most relevant for the nematodes.

Interestingly, temperatures in spring generally appear suitable for both *Caenorhabditis* species, yet nematodes could usually not be isolated during this period, possibly as a result of nematode population dynamics during winter, which are currently unknown. It is possible that during winter the nematodes move to deeper compost layers with more favourable temperatures or die out in these substrates, followed by recolonization in spring or summer. Our sampling scheme did not allow us to evaluate these alternatives, because sampling depth was kept constant at up to 15 cm over the 18 month-period to ensure comparability of sampling results across time.

Humidity and rain were additionally expected to affect *Caenorhabditis* distributions. Moisture was shown for other nematode species to influence migratory dynamics [28]. Moisture may also be important to prevent desiccation, a known cause of death for non-dauer stages of *C. elegans* under laboratory conditions, and to enhance presence and proliferation of microbial food. For our samples, we were able to identify a significant positive correlation between humidity and *C. elegans* on compost in Münster and between humidity and *C. remanei* on rotten apples in Kiel. Furthermore rain was correlated with *C. elegans* occurrence in compost from Kiel. Humidity may thus have some influence on *Caenorhabditis* distributions. In the future, it would be important to investigate in more detail the consequences of humidity variations, not only in the air as measured by us, but also at much smaller scale directly in the substrates.

In contrast, we could not identify a statistically relevant association with pH. This result may be due to wide pH tolerance levels, which were previously reported for *C. elegans* under laboratory conditions [29].

### Additional factors

Biotic factors are likely to play a central role in shaping *Caenorhabditis* distributions in nature. In this context, substrate availability, occurrence of either food or pathogenic microbes, and also the presence of competitors and predators should be of particular importance. This is supported by the recent observation of substantial natural variation in putative immunity genes across French *C. elegans* populations [30]. Within the framework of our study, these factors were not directly assessed and we will briefly discuss their potential importance below.

One basic requirement for proliferation is the availability of suitable substrates. The quality of compost depends on the addition of new material that is left to rot and rotting organic material is only available for a limited time before its complete disintegration. It is known that fresh compost and rotting fruits contain higher numbers of *C. elegans* compared to older compost or soil [12]. Regular compost mixing may thus alter availability of suitable substrates. For the considered composts, this factor may have had only a minor influence during the sampling period, because composts had either not been mixed at all (Münster farming museum), or only once in May 2012 (Roxel private garden); or three times (October 2011, May 2012, and October 2012; Kiel botanical garden), where it did not seem to have an obvious effect on nematode abundance (Figure 1). Substrate availability may also play an important role in winter, where usually little plant material is added from gardens for decomposition, and it could also be influenced by other animals such as the sheep in the Münster farming museum. At this location, sheep were maintained continuously on the same meadow, from which we collected apples and where they most likely ate fruits, possibly limiting the time during which rotting apples were available as substrate. This may explain why *C. elegans* was not found on Münster apples in 2011 but only in 2012 and, in this case, only on apples separated from the sheep by a fence, although at the same time, this factor did not prevent high *C. remanei* prevalence on apples in both years.

The presence and diversity of natural microbes are most likely of central importance for worm fitness, either as food or pathogens. Their exact role under natural conditions is currently unexplored. At least under laboratory conditions, different food bacteria were previously shown to vary in their effect on nematode reproductive rates [31], while *C. elegans* also shows significant natural variation in its resistance towards the possibly naturally associated pathogens *Bacillus thuringiensis* or *Serratia marcescens*[32,33]. Similarly, as indicated above, there may be substantial competition between different *Caenorhabditis* species and also between these and other nematodes if these overlap in their ecological niches. Finally, predators and vectors may additionally influence the population biology of natural *Caenorhabditis* populations. We found several invertebrate species to coexist with our *Caenorhabditis* nematodes such as mites, isopods, springtails, flies, spiders, earwigs, beetles, ants, snails and slugs (unpublished data). Some of these are likely predators of *Caenorhabditis* worms such as the nematophagous mites [22]. Others have been proposed to act as vectors, enhancing nematode migration [15]. Humans may additionally influence nematode distributions by providing stable microbe-rich habitats, by actively distributing worms through transport of organic material, and also by destruction of the more natural habitats, thus enhancing their presence in anthropogenically influenced locations.

## Conclusion

*C. elegans* and *C. remanei* can be found regularly in compost or rotten apples in North German locations with *C. elegans* being more prevalent in compost and *C. remanei* in apples, possibly as a consequence of competition between the two taxa. The exact range of factors influencing the species' distributions are yet unknown. Our analysis suggests at least some influence from certain abiotic factors like humidity and rain. It is likely that biotic factors are additionally a major determinant of *Caenorhabditis* prevalence. Their detailed analysis, especially the influence of different food microbes, pathogens, predators, and vectors, deserves particular attention in the future.

## Competing interests

The authors declare that they have no competing interests.

## Authors’ contributions

CP carried out the sampling, processed the samples, isolated worms and drafted the manuscript. PD helped with the sampling and statistical analysis. EAS helped with sampling, processing of the samples and abiotic measurements. SP helped with sampling and abiotic measurements. HS carried out the statistical analysis, helped to draft the manuscript and supervised the project. All authors read and approved the final manuscript.

## Supplementary Material

Additional file 1: Figure S1 Exemplary results for species-diagnostic PCRs for (A) *C. remanei* and *C. briggsae*, (B) *C. elegans* (two diagnostic PCRs). The order of samples is always DNA of *C. elegans* (1), *C. remanei* (2), *C. briggsae* (3), and negative control (4). The outer lanes contain a 100 bp-DNA ladder. Lenghts of 1000 bp and either 300 bp or 150 bp are indicates on the far left. The specificity of the different targets of the diagnostic PCRs are indicated at the top of each panel (see also Table 1 of the main text). Click here for file

Additional file 2: Figure S2 Locations and samples in the botanical garden in Kiel. Rotten apples were mainly collected from an apple compost (A). Compost was sampled from three big heaps (B) containing plant material in different stages of degradation. Samples were collected in plastic bags and placed on plates in the laboratory (C). *C. elegans* was found in different compost samples (D-F) while *C. remanei* was mainly found on rotten apples (G, H). Click here for file

Additional file 3: Figure S3 The farming museum in Munster. A compost heap (A) and apple trees (B) are located on the same meadow. Sheep have access to both, compost and apples. Rotten apples have been collected below the trees. *C. elegans* was found in different compost samples (C, D) and rarely on rotten apples (E). *C. remanei* was mainly found on rotten apples (F-H) and only a few times on compost. Click here for file

Additional file 4: Figure S4 Compost samples from a private garden in Roxel. The compost is stored in three mesh cages (A). The cages contain plant material in different stages of decomposition (B-G). *C. elegans* has been found regularly in compost samples from all cages. Other *Caenorhaditis* species were rarely found. Click here for file

Additional file 5: Table S1 Complete list of newly isolated *Caenorhabditis* strains. The list contains information on strain name, internal strain code, independence of samples, exact location of origin, substrate from which nematodes were isolated, and date of sampling. Click here for file

Additional file 6: Table S2 Detailed overview of prevalence of *C. elegans* and *C. remanei* across the three North German locations. The list provides information on number and proportion of samples per substrate, location, and month. Click here for file
