# Supplementary figures and images for: The prevalence of Caenorhabditis elegans across 1.5 years in selected North German locations: the importance of substrate type, abiotic parameters, and Caenorhabditis competitors
Source: BMC Ecol. 2014 Feb 6;14:4. doi: 10.1186/1472-6785-14-4 (PMC3918102; doi:10.1186/1472-6785-14-4)

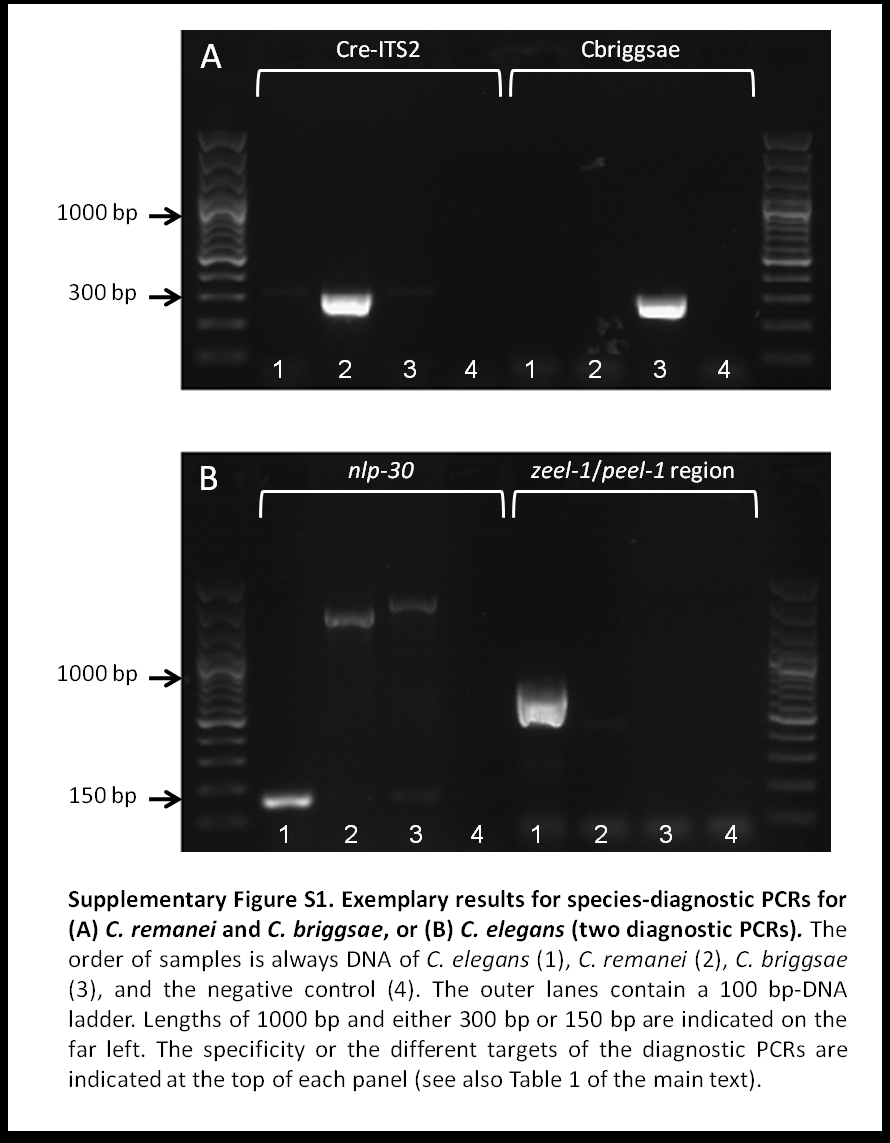

Supplement: Additional file 1: Figure S1 — Exemplary results for species-diagnostic PCRs for (A) C. remanei and C. briggsae, (B) C. elegans (two diagnostic PCRs). The order of samples is always DNA of C. elegans (1), C. remanei (2), C. briggsae (3), and negative control (4). The outer lanes contain a 100 bp-DNA ladder. Lenghts of 1000 bp and either 300 bp or 150 bp are indicates on the far left. The specificity of the different targets of the diagnostic PCRs are indicated at the top of each panel (see also Table 1 of the main text). [file 1472-6785-14-4-S1.tiff]
