# Supplementary material for: The prevalence of Caenorhabditis elegans across 1.5 years in selected North German locations: the importance of substrate type, abiotic parameters, and Caenorhabditis competitors
Source: BMC Ecol. 2014 Feb 6;14:4. doi: 10.1186/1472-6785-14-4 (PMC3918102; doi:10.1186/1472-6785-14-4)

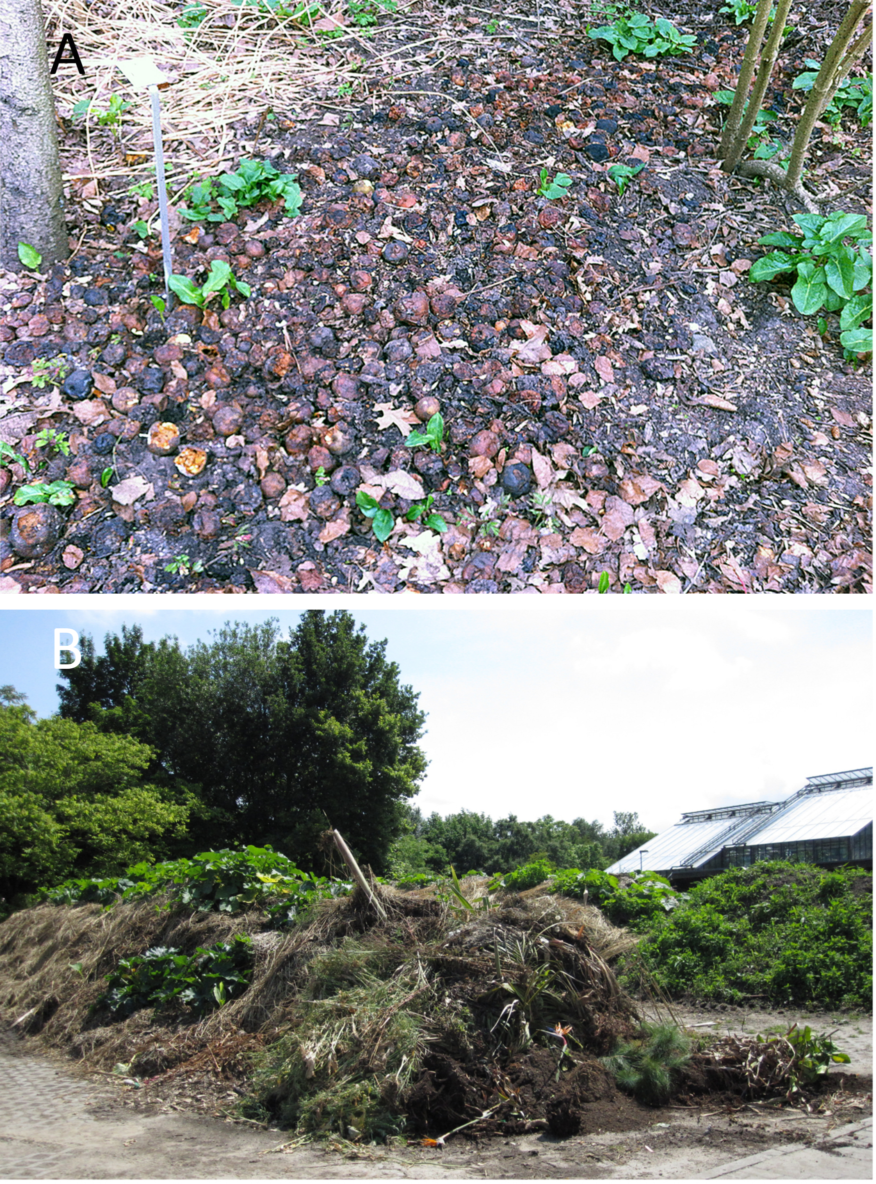

Figure S2. Locations and samples in the botanical garden in Kiel. Rotten apples were mainly collected from an apple compost (A). Compost was sampled from three big heaps (B) containing plant material in different stages of degradation. Samples were collected in plastic bags and placed on plates in the laboratory (C). *C. elegans* was found in different compost samples (D – F) while *C. remanei* was mainly found on rotten apples (G, H).

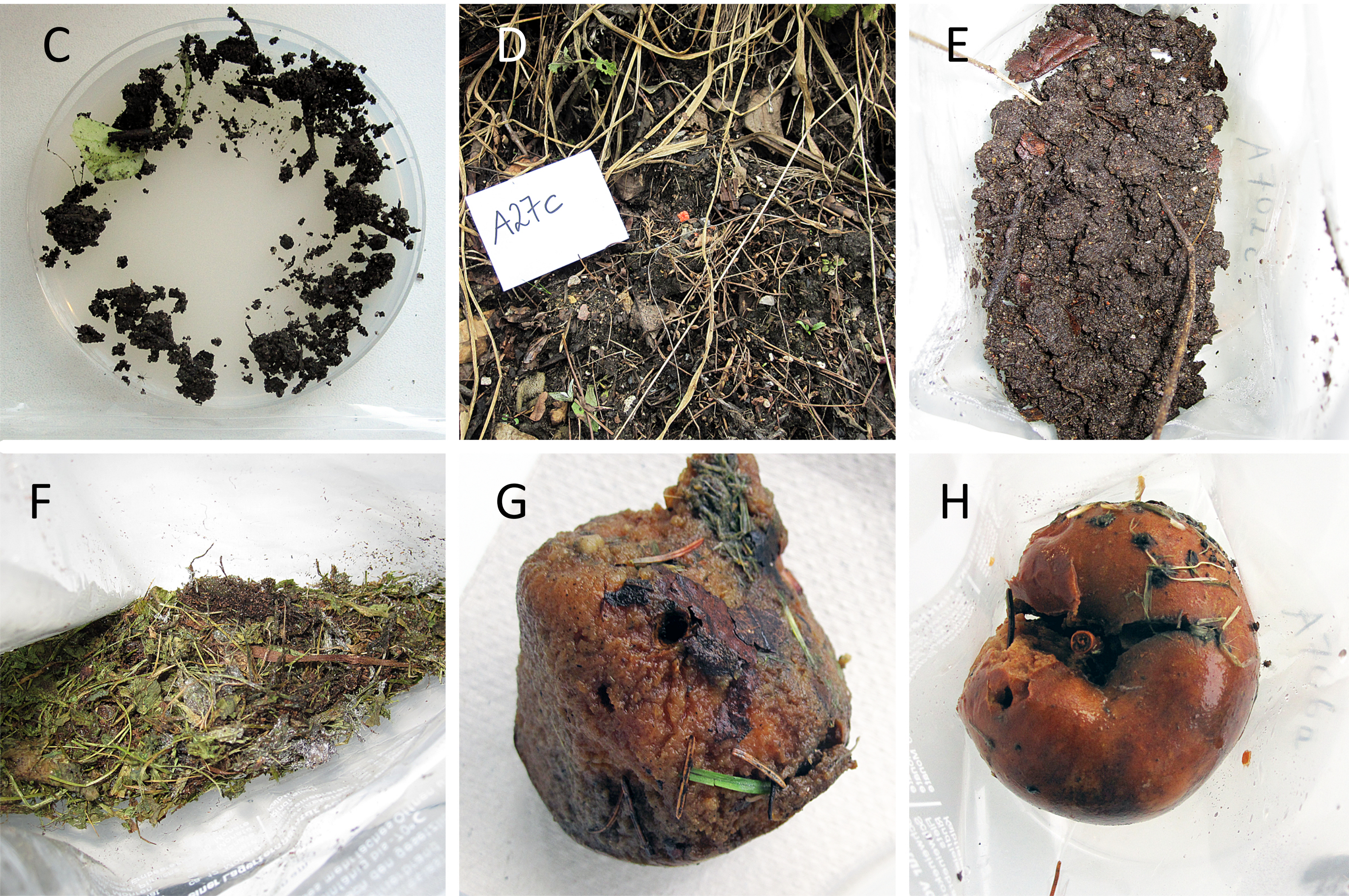

Supplement: Additional file 2: Figure S2 — Locations and samples in the botanical garden in Kiel. Rotten apples were mainly collected from an apple compost (A). Compost was sampled from three big heaps (B) containing plant material in different stages of degradation. Samples were collected in plastic bags and placed on plates in the laboratory (C). C. elegans was found in different compost samples (D-F) while C. remanei was mainly found on rotten apples (G, H). [file 1472-6785-14-4-S2.pdf]
