# Supplementary material for: The prevalence of Caenorhabditis elegans across 1.5 years in selected North German locations: the importance of substrate type, abiotic parameters, and Caenorhabditis competitors
Source: BMC Ecol. 2014 Feb 6;14:4. doi: 10.1186/1472-6785-14-4 (PMC3918102; doi:10.1186/1472-6785-14-4)

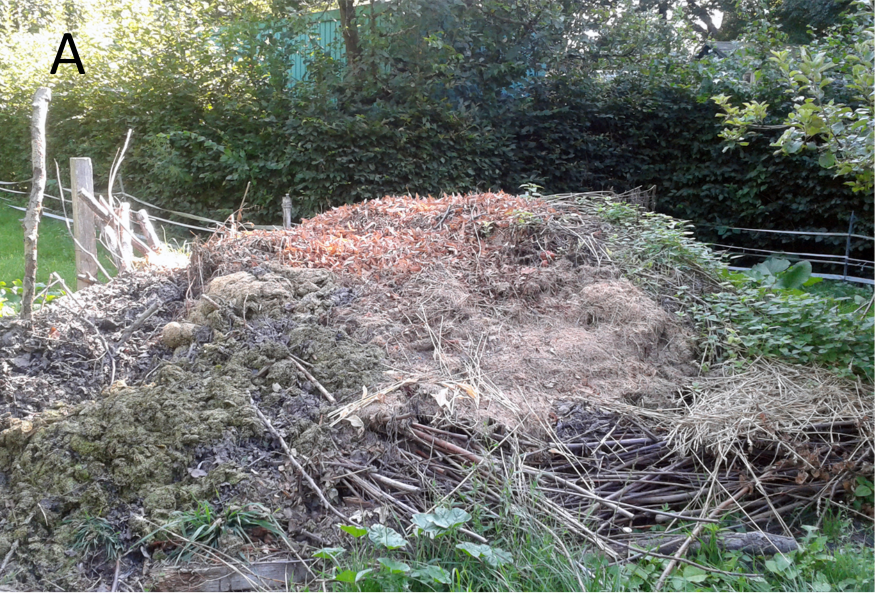

Figure S3. The farming museum in Münster. A compost heap (A) and apple trees (B) are located on the same meadow. Sheep have access to both, compost and apples. Rotten apples have been collected below the trees. *C. elegans* was found in different compost samples (C, D) and rarely on rotten apples (E). *C. remanei* was mainly found on rotten apples (F – H) and only a few times on compost.

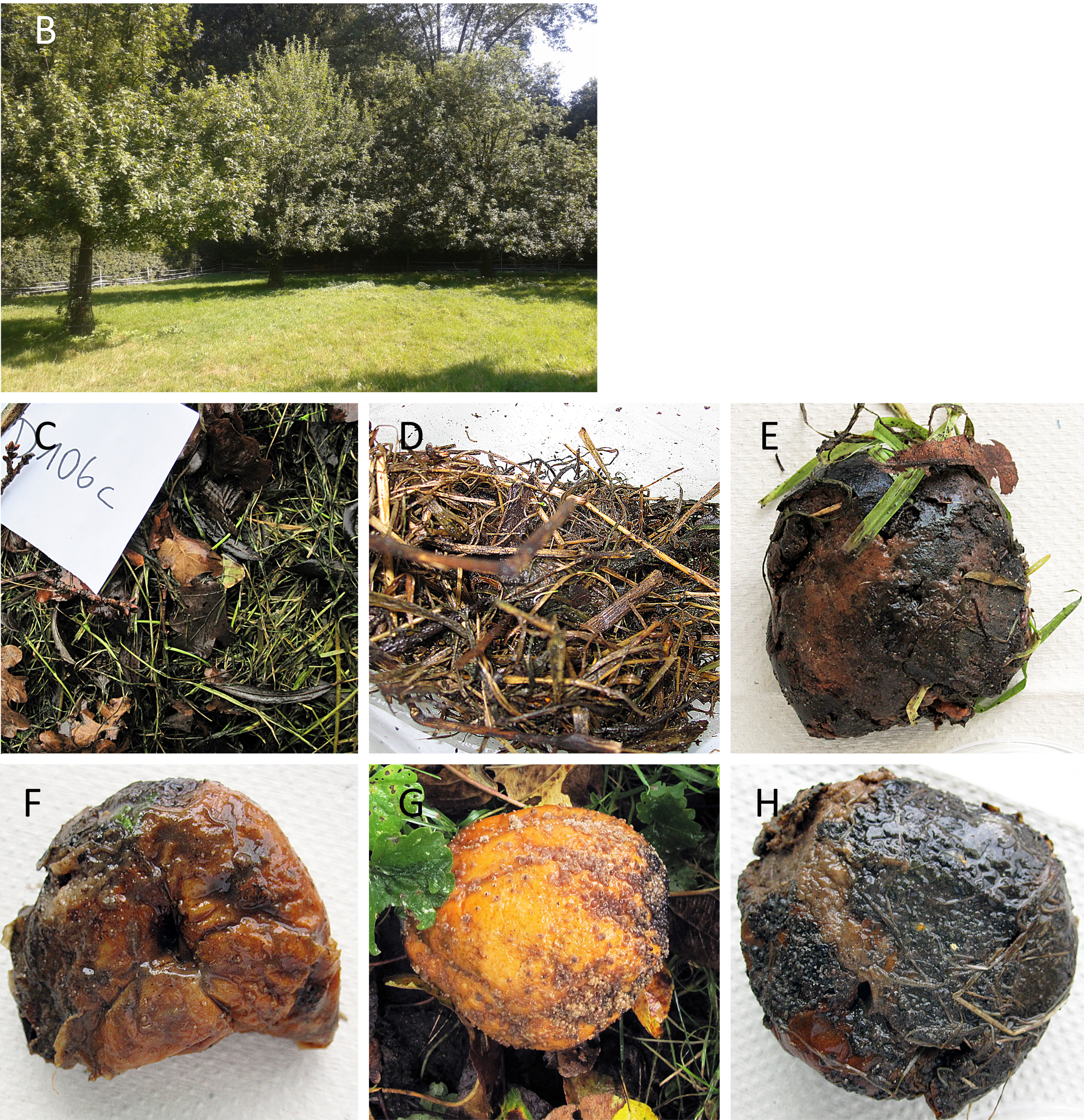

Supplement: Additional file 3: Figure S3 — The farming museum in Munster. A compost heap (A) and apple trees (B) are located on the same meadow. Sheep have access to both, compost and apples. Rotten apples have been collected below the trees. C. elegans was found in different compost samples (C, D) and rarely on rotten apples (E). C. remanei was mainly found on rotten apples (F-H) and only a few times on compost. [file 1472-6785-14-4-S3.pdf]
