# Supplementary material for: The prevalence of Caenorhabditis elegans across 1.5 years in selected North German locations: the importance of substrate type, abiotic parameters, and Caenorhabditis competitors
Source: BMC Ecol. 2014 Feb 6;14:4. doi: 10.1186/1472-6785-14-4 (PMC3918102; doi:10.1186/1472-6785-14-4)
Supplement: Additional file 4: Figure S4 — Compost samples from a private garden in Roxel. The compost is stored in three mesh cages (A). The cages contain plant material in different stages of decomposition (B-G). C. elegans has been found regularly in compost samples from all cages. Other Caenorhaditis species were rarely found. [file 1472-6785-14-4-S4.pdf]

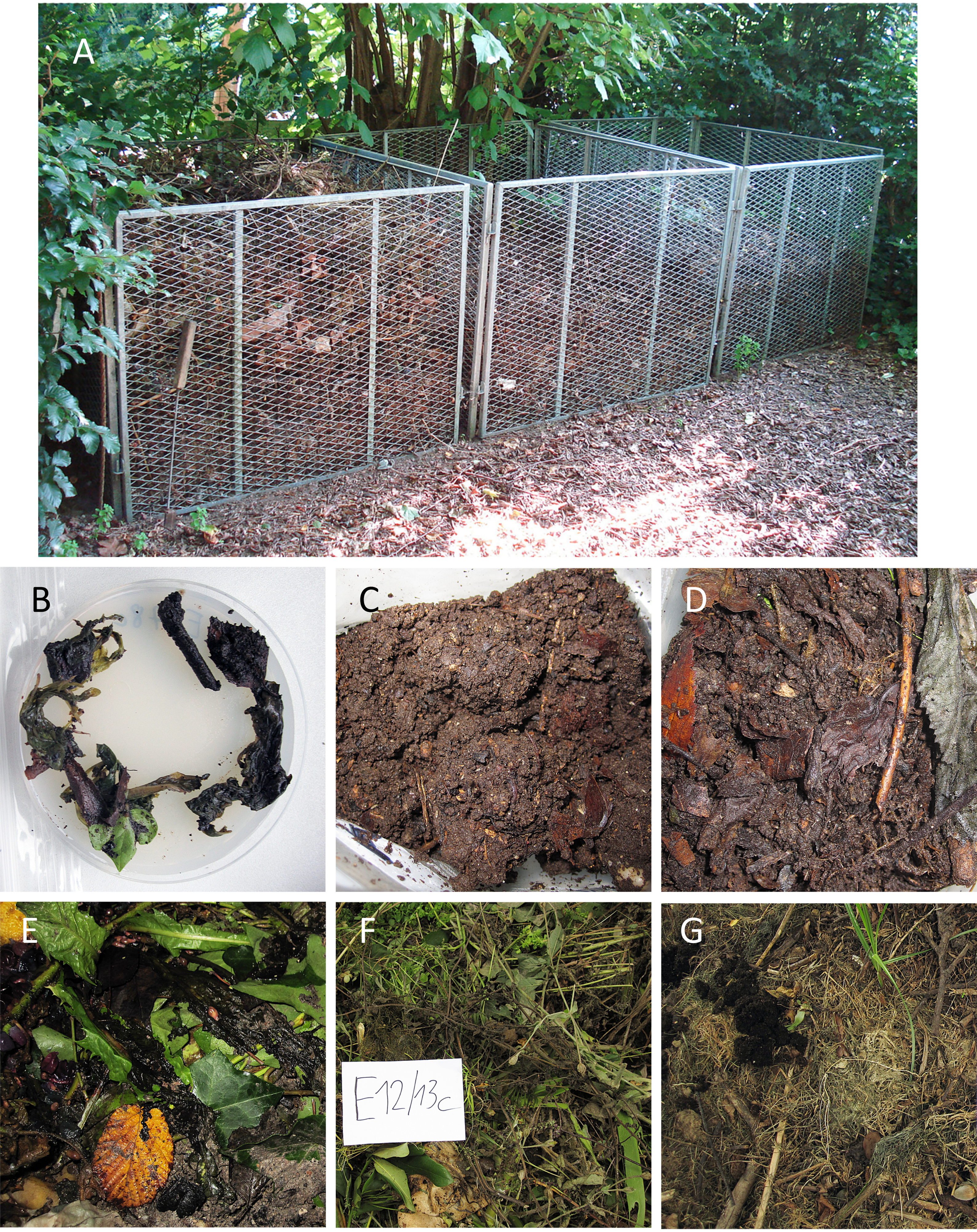

Figure S4. Compost samples from a private garden in Roxel. The compost is stored in three mesh cages (A). The cages contain plant material in different stages of decomposition (B – G). *C. elegans* has been found regularly in compost samples from all cages. Other *Caenorhabditis* species were rarely found.
